# Immediate reuse of patch-clamp pipettes after ultrasonic cleaning

**DOI:** 10.1038/s41598-024-51837-7

**Published:** 2024-01-18

**Authors:** Kevin Jehasse, Jean-Sébastien Jouhanneau, Sophie Wetz, Alexander Schwedt, James F. A. Poulet, Peter Neumann-Raizel, Björn M. Kampa

**Affiliations:** 1 Systems Neurophysiology, Institute of Biology II, RWTH-Aachen University, Aachen, Germany; 2 Central Facility for Electron Microscopy, RWTH-Aachen University, Aachen, Germany; 3 JARA BRAIN, Institute of Neuroscience and Medicine (INM-10), Forschungszentrum Jülich, Jülich, Germany; 4 Research Training Group 2610 InnoRetVision, RWTH-Aachen University, Aachen, Germany; 5 Max Delbrück Center for Molecular Medicine in the Helmholtz Association (MDC), Berlin, Germany; 6 Neuroscience Research Center, Charité-Universitätsmedizin Berlin, Berlin, Germany; 7 Luigs & Neumann GmbH, Ratingen, Germany

**Keywords:** Cellular neuroscience, Patch clamp

## Abstract

The patch-clamp technique has revolutionized neurophysiology by allowing to study single neuronal excitability, synaptic connectivity, morphology, and the transcriptomic profile. However, the throughput in recordings is limited because of the manual replacement of patch-pipettes after each attempt which are often also unsuccessful. This has been overcome by automated cleaning the tips in detergent solutions, allowing to reuse the pipette for further recordings. Here, we developed a novel method of automated cleaning by sonicating the tips within the bath solution wherein the cells are placed, reducing the risk of contaminating the bath solution or internal solution of the recording pipette by any detergent and avoiding the necessity of a separate chamber for cleaning. We showed that the patch-pipettes can be used consecutively at least ten times and that the cleaning process does not negatively impact neither the brain slices nor other patched neurons. This method, combined with automated patch-clamp, highly improves the throughput for single and especially multiple recordings.

## Introduction

Patch-clamp recording is a widely-used and powerful technique to study single-cell electrophysiology, especially in neuroscience. It led to the characterization of several aspects of neuronal physiology, in vitro and in vivo, such as ion channel activity underlying action potential^1,2^, intrinsic excitability^3,4^, synaptic integration^5,6^, plasticity^7,8^ and network activity^9,10^. Patch-clamp also allows recordings in the different compartments of neurons^11–13^ including very distant dendritic branches or axonal sections^14^. In the *whole-cell* configuration, it is possible to dialyze cells with fluorescent dyes for morphological reconstructions and to collect the cytoplasm to analyze single-cell transcriptomic profiles^15,16^. Although the patch-clamp technique is well suited to characterize the heterogeneity of neurons in the brain, it is highly laborious and time-consuming with variable success rates resulting in a low-throughput. To overcome this issue, engineering advancements managed to automate patch-clamp in vitro and in vivo^17–19^. When automated, the software is able to track the patch pipette and individual neuron positions in order to approach and record them. While it reduces the human interaction for the recording, it is still required to manually change the patch pipette after each attempt.

To obtain a successful recording, one crucial parameter is to have a clean patch-clamp pipette tip filled with a filtered internal solution^20^. In this condition, there is a high chance to form a seal of high-resistance (≥ 1 GΩ, which is called “gigaseal”) with the membrane of the neuron. After each successful or failed attempt, the used pipette has to be manually changed. Therefore, the human factor can be a big issue when it comes to multiple recordings because of vibrations from the exchange that can affect the seal of other pipettes attached to neurons in the context of neuronal network characterization. It can also be an issue for in vivo patch-clamp studies as the manual pipette replacement could disrupt the animal and the following recordings. To prevent that, an automated method for cleaning patch pipettes has recently been developed^21,22^. This approach consists of immersing the tip of used patch-clamp pipettes in a separate chamber containing the detergent Alconox while applying cycles of positive and negative pressure to the pipette tip. Coupled with automated patch-clamp^23–25^, the throughput of patch-clamp recording is significantly increased. However, the necessity of a separate bath cleaning chamber and the risk of contaminating the recording bath solution nor pipette internal solution with detergent reducing the usability of this cleaning method.

**Figure 1:**
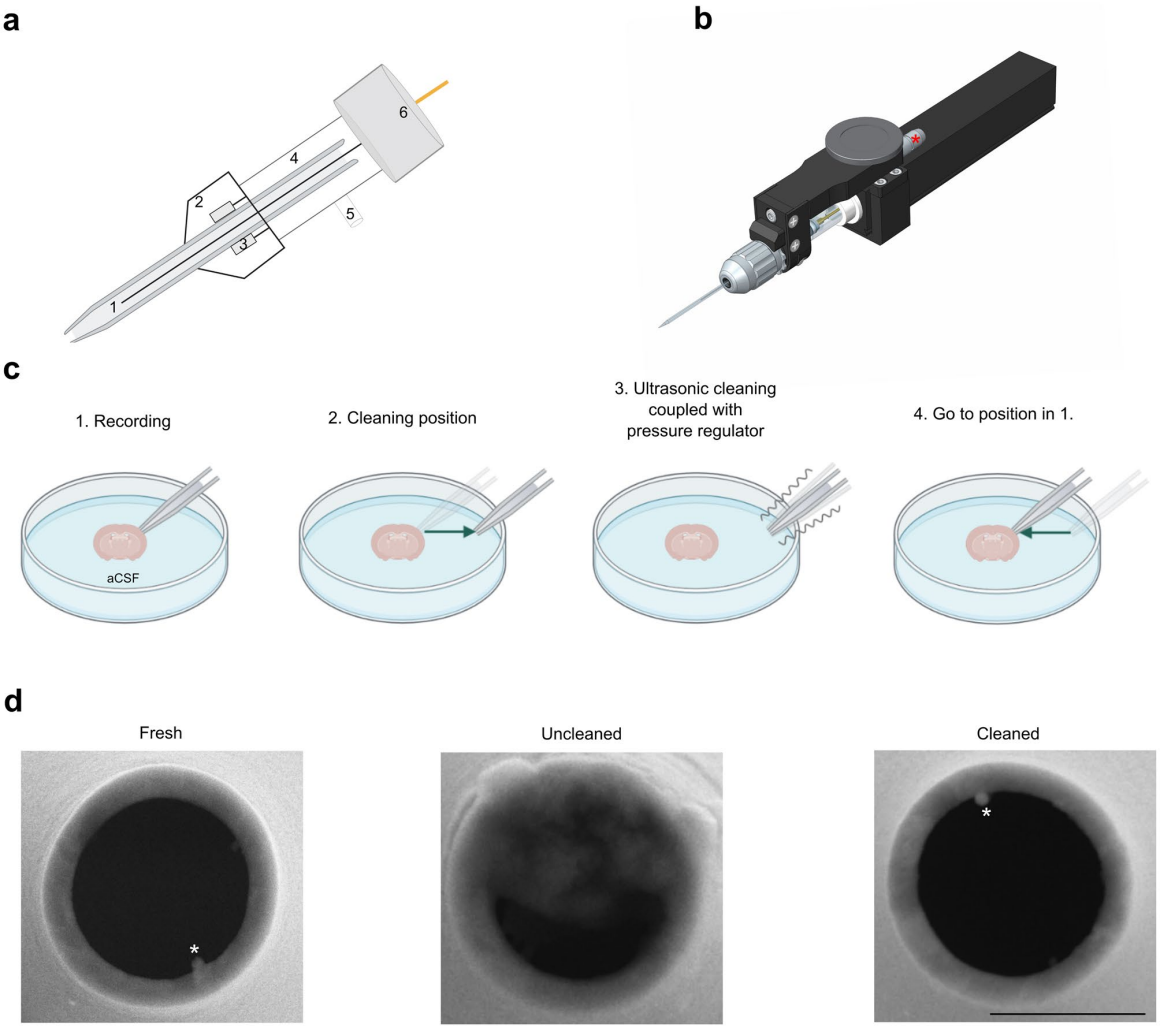
Ultrasonic cleaning is performed by a piezo coupled to a pressure regulator. **(a)** Schematic representation of the piezo-mounted headstage. (1) Patch-clamp pipette filled with internal solution and the AgCl wire. (2) Pipette holder tightener. (3) Piezo inside the pipette holder tightener in direct contact with patch-clamp pipette. (4) Pipette holder. (5) Input for pressure control. (6) Input to headstage. **(b)** Overview of the ultrasonic cleaning mounted headstage. Asterisk shows the input for ultrasonic control. **(c)** When the recording is finished (1), the cleaning sequence can be launched. The manipulator put the tip to a pre-defined (in a radius of ideally 5 mm in all plans) cleaning position (2) where the sonification coupled with cycle of high positive and negative pressure can occur (3). Once the procedure is finished, the manipulator put the tip back to its initial parking position (4) before the cleaning was launched. **(d)** Scanning electron microscopy (SEM) images of tips from a fresh pipette, an uncleaned pipette after reaching *whole-cell* and a cleaned pipette with sonification after reaching *whole-cell*. The tip of the ultrasonic cleaned pipette is similar to the fresh one, while the uncleaned tip is contaminated with cellular debris. The star (asterisk) indicates the glass filament inside the pipette. Scale bar is x = 1 µm.

Since patch-clamp pipettes are made of borosilicate glass and can also be cleaned by sonification, we developed a novel cleaning system based on that method preventing the use of detergent. The sonification is performed by a piezo-element mounted on the recording headstage and connected to a pressure control system (Fig. 1a, b). The ultrasonic cleaning is performed in artificial cerebrospinal fluid (aCSF) and allows the reuse of the same patch-clamp pipette at least ten times without affecting the recording quality. We also showed that the cleaning procedure within the same bath does not affect the stability of seal of other patch-clamp pipettes with their respective neurons. Therefore, ultrasonic cleaning is a powerful improvement offering significant advantages in particular for multiple simultaneous patch-clamp recordings.

## Results

### Cleaning efficiency

Before approaching the patch-clamp pipette to the brain slice, a “cleaning position” (between 1 and 5 mm from the slice) was defined to safely clean the pipette without affecting the sample. This is determined by the micromanipulator control panel. When “cleaning” is selected, the micromanipulator automatically goes to the saved XYZ coordinates (Fig. 1b, c). The piezo mounted on the headstage performs the sonification of the patch-clamp pipettes by making it oscillate at 40 kHz. To help removing the membrane residue inside the pipette, the sonification is coupled with a cycle of high positive and negative pressure similar to the protocol used for cleaning in detergent^21^. After the cleaning procedure the patch-clamp pipette can be placed into a “parking position” which corresponds to a determined XYZ distance to its original place. This prevents any damage of the slice by the automatic replacement of the pipette and to freely move to find another neuron to record. First, we used scanning electron microscopy to confirm that ultrasonic cleaning in aCSF allows the reuse of pipettes, as the tip of a cleaned one is similar to a fresh one (Fig. 1d).

**Figure 2:**
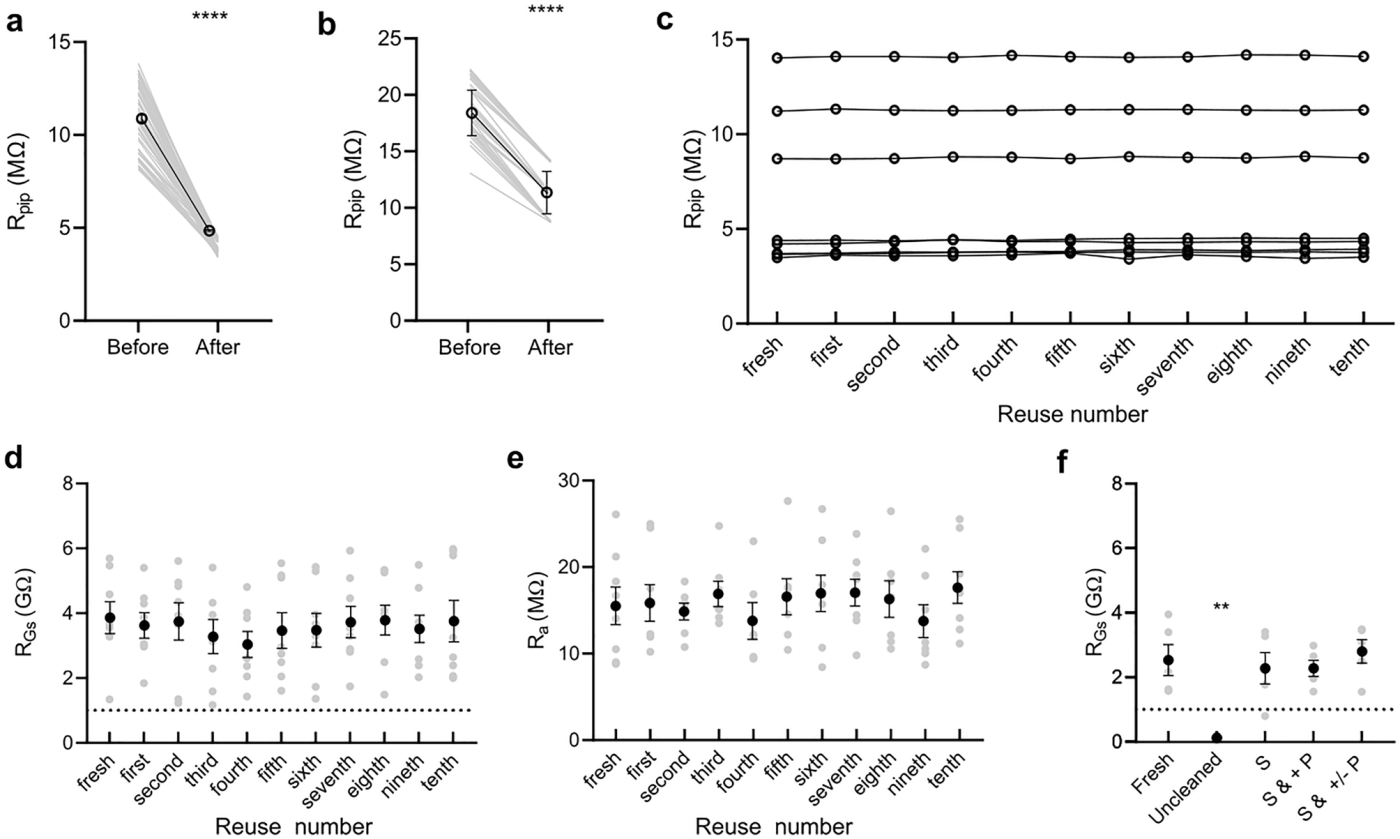
Ultrasonic cleaning allows reusing pipettes for multiple consecutive recordings. **(a,b)** Change in R_pip_ before and after cleaning of the tip of low resistance **(a)** and high resistance **(b)** pipettes. Single values are represented in grey, while mean ± SEM values are represented in black. **(c)** Evolution of R_pip_ over several cleaning stages. **(a–c)** Ultrasonic cleaning can clean the tip as R_pip_ is recovered after each cycle. **(d)** Patch-clamp pipette can be successfully reused at least ten times and R_GS_ is successfully reached. Single values are represented in grey, while mean ± SEM values are represented in black. **(e)** R_a_ remains unaffected after each cleaning cycle, indicating that the tip is cleaned and the pipette can be reused for successful patch-clamp recordings. Single values are represented in grey, while mean ± SEM values are represented in black. **(f)** Ultrasonic cleaning can allow pipette to be reused to reach a gigaseal. S sonification only, S & + P sonification combined with positive pressure only, S & + /− P sonification combined with alternance of positive and negative pressure.

For each attempt, the pipette resistance (R_pip_) was monitored once a *whole-cell* recording attempt was made, and compared before and after the ultrasonic cleaning (Fig. 2a) to assess the efficiency of the procedure. We used pipettes with R_pip_ between 3 and 5 MΩ (Fig. 2a) and between 8 and 15 MΩ (Fig. 2b) to test the efficiency of ultrasonic cleaning on patch-pipettes with regular tip size and sharp tip size regularly used, respectively for somatic recordings and for dendritic recordings^26^ or in vivo patch-clamp. As expected, R_pip_ was reduced after sonification (before: 11.08 ± 0.22 MΩ,; after: 3.98 ± 0.05 MΩ, n = 55 attempts, p < 0.0001, for low resistance pipettes; before: 18.78 ± 0.41 MΩ, after: 11.38 ± 0.39 MΩ, n = 33 attempts, p < 0.0001, for high resistance, two-tailed Wilcoxon signed-rank test, Fig. 2a, b). Over ten cleaning cycles, R_pip_ remained constant (p = 0.6454 for fresh vs tenth cycle, n = 8, two-tailed Wilcoxon signed-rank test; Fig. 2c). We also performed over ten cycles of cleaning to test the limit of ultrasonic cleaning (Supplementary Fig. 1). We could manage to reach gigaseal (Supplementary Fig. 1a) and *whole-cell* configuration with a good access (Supplementary Fig. 1b) after nearly 30 cycles, suggesting that pipettes can be reuse indefinitely with ultrasonic cleaning. These data show that ultrasonic cleaning coupled with alternating high and low pressure can successfully remove remaining membrane residues and repeatedly over at least ten attempts.

Next, we evaluated the quality of recordings by monitoring the resistance of gigaseal (R_GS_) and access resistance (R_a_). R_GS_ was obtained before breaking in *whole-cell*, when the value was steady. We managed to reach gigaseal in 88 neurons and *whole-cell* configuration in 81 neurons with 8 pipettes (92.05% success rate) that were cleaned ten times and we observed no effect of ultrasonic cleaning on R_GS_ (p = 0.988, Friedman test; Fig. 2d) and R_a_ (p = 0.8192, Kruskal–Wallis test; Fig. 2e). Lastly, we evaluated the success rate of reach R_GS_ with and without sonification combined with pressure (Fig. 2f). We could not reach the gigaseal with uncleaned pipette (fresh vs uncleaned: p < 0.046, Friedman test followed by Dunn’s multiple comparisons test; Fig. 2f), while cleaning the allowed to reach gigaseal. We tested cleaning with sonification only (S), sonification combined with positive pressure only (S & + P) and sonification combined with alternance of positive and negative pressure (S & + /− P). Even though sonification only allows the reuse of patch-clamp pipettes (fresh versus S, S & + P and S & + /− P p > 0.99, Friedman test followed by Dunn’s multiple comparisons test; Fig. 2f) we failed to reach gigaseal one time (80% success rate), while we always succeeded reaching the gigaseal when the pressure was added. These results show that with the novel sonification method patch-clamp pipettes can be reused at least 10 times for successful *whole-cell* recordings.

Since this cleaning method gives promising results for in vitro recordings, we then tried to reuse patch-clamp pipette for in vivo recordings (Supplementary Fig. 2). While the pipette can be cleaned after sonification in the recording solution (Supplementary Fig. 2b), we did not manage to reach a sufficient R_GS_ (Supplementary Fig. 2c). Nevertheless, ultrasonic cleaning in Tergazyme allowed the reuse of pipette as a sufficient R_GS_ was obtained in all trials. The use of Tergazyme did not affect neither the neurons excitability nor their responses to airpuffs (Supplementary Fig. 2d–j).

### Effect of sonification on cell survival

**Figure 3:**
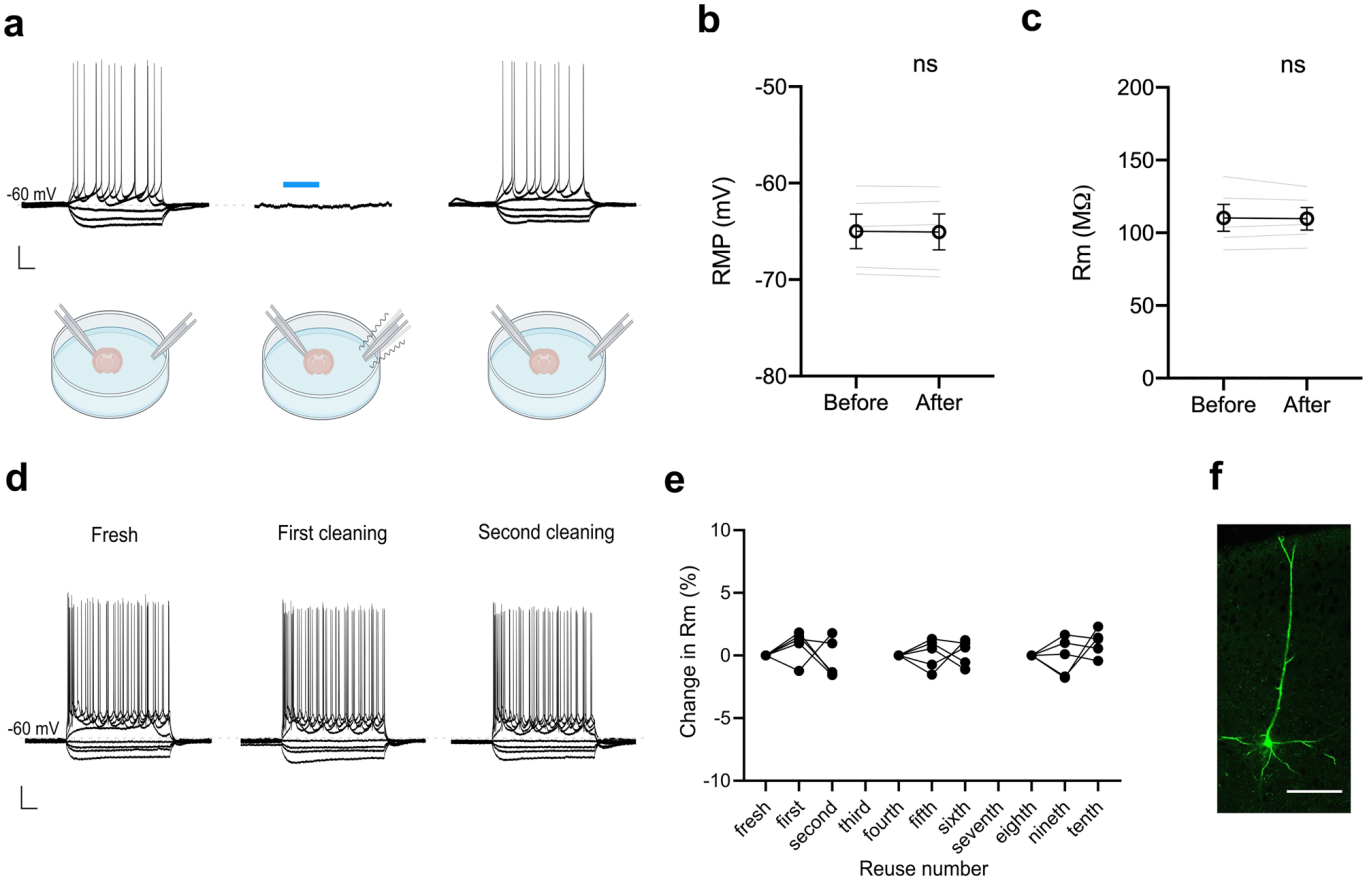
Ultrasonic cleaning is not harmful for neurons. **(a)** Example of a neuron being recorded before (left), during (middle) and after (right) ultrasonic cleaning of a patch-clamp pipette inside the same recording chamber. Scale bar is y = 20 mV (all panels) and x = 100 ms (left and right) and 20 s (middle). Blue bar corresponds to the moment when the non-recording tip is being cleaned. **(b,c)** The cleaning procedure does not interfere with simultaneous patch-clamp recordings as RMP and R_m_ of the recorded neurons remain unaffected. Single values are represented in grey, while mean ± SEM values are represented in black. **(d)** Example of a neuron being successfully recorded three consecutive times with the patch-clamp pipette at the soma after ultrasonic cleaning. Scale bar is y = 20 mV and x = 100 ms. **(e)** Changes in R_m_ from neurons being recorded several times at different cleaning stages. First attempts of recording neurons were performed with a fresh pipette, after four and eight cleaning sequences. **(f)** Post-hoc staining of a neuron from **(d)**. Scale bar is x = 80 µm.

We next tested whether ultrasonic cleaning could affect neuronal survival within the same chamber. In the context of simultaneous paired recordings, it is crucial that cleaning one pipette tip does not affect other neurons maintained in *whole-cell* by the patch-clamp pipettes. At 1 mm from the slice, we observed a resonance effect on the recording pipette while the other one was being cleaned (Supplementary Video 1), therefore harming the cell being recorded and leading to a loss of the recording. At 5 mm, we did not observe this resonance effect (Supplementary Video 2). In this condition, the resting membrane potential (RMP) of neurons being recorded simultaneously remained stable during the cleaning process (before: -65.0 ± 1.8 mV; after: -65.1 ± 1.9 mV, n = 5, p = 0.5625, two-tailed Wilcoxon signed-rank test; Fig. 3a, b) and membrane resistance (R_m_) is not affected (before: 110.3 ± 9.3 MΩ; after: 109.7 ± 7.7 MΩ, n = 5, p > 0.99, two-tailed Wilcoxon signed-rank test; Fig. 3a, c). Finally, in several cases, we successfully recorded the same neuron three times with the same patch-clamp pipette at the soma after being cleaned at different stages (n = 15 in total, 5 neurons per group starting at “fresh” pipette, after “fourth” and “eighth” cleaning sequences) and filled it with biocytin for confirmation (Fig. 3d, e). We monitored the change in R_m_ and found that it remained stable for all 3 attempts which suggests that the internal solution is not altered during the cleaning procedure (p = 0.54, Friedman test). Moreover, the post-hoc reconstructed morphology did not show any sign of neuronal damage (Fig. 3f). Altogether, these data show that ultrasonic patch-clamp pipette cleaning does not harm neurons situated within the same bath chamber.

## Discussion

The patch-clamp technique is a very powerful method but it is laborious and the success rate is not high as it depends on the skills of the experimenter and the quality of the sample tested. In order to increase its throughput, automatization of the patch-clamp technique has recently been the focus of engineering. While there have been several successful developments to automate the whole procedure^17,19,23^ it is crucial to overcome the changing of patch-clamp pipette. Others have shown that it is possible to clean the tips using either bleach^27^ or detergent combined with automated pressure control^21,28^. However, even if not detected, there might be some trace left of these chemicals inside the patch-clamp pipette that can be harmful for the cells. We developed an ultrasonic cleaning system coupled with automated pressure control, which allows the tips to be cleaning in a physiological solution and is less likely to harm the cells. In addition, being able to clean the pipette tip in standard aCSF, our method offers the possibility to avoid using a second compartment. While this would also be convenient for in vivo patch-clamp recordings, our results showed that cleaning the pipette with sonification in the recording solution is not optimal yet, and we needed a second bath containing Tergazyme to be able to reuse the pipette as shown by others^21^. This could be explained by the fact that, after in vivo recordings, the recording pipette is contaminated with cellular debris and blood cells and sonification alone is not powerful enough to clean the tip of the pipette for reuse. However, sonification has also been shown to be useful for in vivo patch-clamp recordings in combination with a separate cleaning bath solution. And coupled with recently automated in vivo patch-clamp recordings^17^, the ultrasonic cleaning system coupled with automated pressure control will increase the throughput of these recordings both in vitro and in vivo and make the method also more accessible.

Cleaning patch-clamp pipettes remains to this day one of the key elements to automate patch-clamp recordings. In the context of drug discovery, automated patch-clamp experiments are essential to explore the pharmacology of ion channels. Although we did not investigate the effect of ultrasonic cleaning on ion channels, Kolb and colleagues showed that GABA receptors pharmacology is not affected with pipettes being cleaned in Alconox^21^. With these observations combined with ours, showing that ultrasonic cleaning does not affect the physiological properties of the recorded neurons, it can be assumed that our method is not affecting the pharmacology of ion channels either. Repeated recordings from the same neuron with the same pipette cleaned in between consecutive recordings still resulted in the same membrane potential and input resistance. This shows that also the internal solution in the recording pipette is not affected or diluted by the cleaning process. However, it is important to note that such approach is not to be considered in the context of patch-seq, as the pipette has to be removed once the cytoplasm has been taken inside for RNA-seq^16^.

Regarding the longevity of pipettes, we did not investigate how many times pipettes can be used before replacing it with a fresh one. Kolb and colleagues showed that it is not possible to reuse the same pipette indefinitely^21^. We suspect that our approach will allow reusing the same pipettes for a limited time, as internal solution contains chemicals that degrade when not refrigerated (e.g. ATP, GTP). Yet, for the time tested in our experiments, we could repeatedly record even from the same pipette neurons for nearly 30 times suggesting that the lifetime of the pipette is not a limiting factor.

In conclusion, we have developed a detergent free cleaning method for patch-clamp pipettes based on sonification coupled with a pressure control system that allows reusing the recording pipettes and improves the throughput for simultaneous recordings and can be implemented for automated patch-clamp systems.

## Methods

### Cleaning procedure

We used a pressure control system (LN-PCS, Luigs & Neumann, Germany) to deliver positive and negative pressure to the patch-pipette. The procedure is set and triggered with a SM10 Remote Control Touch (Luigs & Neumann, Germany).

For the in vitro recordings, the cleaning protocol consisted of 6 steps alternating positive (+ 500 mBar for 3 s) and negative pressures (-300 mBar for 3 s) with total duration of ~ 20 s or one step of positive pressure (+ 500 mBar) for 20 s and the frequency of sonification is set at 40 kHz. The protocol can be edited within the SM10 Remote Control Touch, where pressure and frequency can be adjusted based on the dimension of the patch-clamp pipette used and user preferences. A safe cleaning distance (~ 5 mm from the brain slice) was defined before the beginning of the experiment to prevent any energy transfer from the pipette being cleaned to other pipettes attached to neurons in *whole-cell* configuration.

For the in vivo recordings, the LN-PCS cleaning protocol consisted of six steps alternating between positive pressure (+ 500 mBar for 3 s) and negative pressure (– 300 mBar for 3 s), or five steps alternating between positive pressure (+ 800 mBar for 3 s) and negative pressure (–400 mBar for 1 s), followed by a positive pressure step of 800 mBar for 10 s at 40 kHz. While cleaning the electrode in the bath Ringer’s solutions, we used different sonication frequencies ranging from 20 to 40 kHz. Various cleaning protocols were employed, including 10 steps of positive pressure (800 mBar for 5 s) or starting with a negative pressure step of – 400 mBar for 4 s, followed by eight steps alternating between positive pressure (+ 800 mBar for 5 s) and negative pressure (− 400 mBar for 2 s), then followed by a positive pressure step of + 800 mBar for 10 s. Two cleaning experiments were conducted in Ringer’s outside the recording chamber, and another experiment was conducted in ddH2O outside the recording chamber. Results from these experiments were combined in the ‘Ringer’s’ dataset. For the Tergazyme cleaning experiments, the default cleaning protocol was used: five steps alternating between positive pressure (+ 800 mBar for 3 s) and negative pressure (– 400 mBar for 1 s), followed by a positive pressure step of + 800 mBar for 10 s at 40 kHz.

### Ethics

All procedures were performed with the approval of local authority (LANUV NRW, Germany and the Berlin State Office for Health and Social Affairs (LAGeSo) according to the directive 2010/63/EU) and in accordance with the European Commission and ARRIVE guidelines for animal experiments.

### In vitro electrophysiology

Acute brain slices were obtained as previously described^29,30^: adult C57BL/6 mice of both genders were anesthetized with isofluorane (AbbVie, UK) and decapitated. 300 µm-thick coronal slices were cut with a vibratome (Leica VT1200s) in an ice-cold modified cutting solution containing (in mM): 125 NaCl, 2.5 KCl, 1.25 NaH_2_PO_4_, 25 NaHCO_3_, 25 Glucose, 6 MgCl_2_, 1 CaCl_2_, pH 7.4 (95% O_2_/5% CO_2_ and 310 mOsm/l). Slices were incubated for 30 min at 34 °C in aCSF containing (in mM): 125 NaCl, 2.5 KCl, 1.25 NaH_2_PO_4_, 25 NaHCO_3_, 25 Glucose, 1 MgCl_2_, 2 CaCl_2_, pH 7.4 (95% O_2_/5% CO_2_ and ~ 310 mOsm/l) and recovered at room temperature.

Patch-clamp recordings were performed using a LNscope (Luigs & Neumann, Germany) equipped with a 40× water immersion objective (Zeiss, Germany), infrared-Dodt gradient contrast and a CMOS camera (Chameleion USB 3.0 monochrome Camera, Point Grey, Canada). Patch pipettes (3–15 MΩ) were pulled from borosilicate glass (GB150F-10, Scientific Products GmbH, Germany) with a horizontal puller (P-1000, Sutter Instruments, Novato, CA, USA). The internal solution contained (in mM): 100 K-gluconate, 20 KCl, 10 HEPES, 4 Mg-ATP, 0.3 Na-GTP, 10 Na_2_-phosphocreatine, 0.3% biocytine, pH 7.2 (~ 300 mosm/l). Data were acquired with an EPC-10 USB amplifier (Heka, Lambrecht, Germany) and Patchmaster Next software (Heka, Lambrecht, Germany). Data were digitized at 20 kHz and lowpass filtered at 10 kHz. *Whole-cell* patch-clamp recordings consisted of current steps from −100 to 300 pA in steps of 50 pA for 500 ms. R_m_ was monitored before and after the cleaning procedure of non-recording pipettes. RMP was monitored during the cleaning process to evaluate any impact of the sonification on the stability of recording. R_pip_, R_GS_ and R_a_ were monitored in voltage-clamp mode to evaluate the efficiency of the cleaning procedure. Change in R_m_ was calculated following this formula:

$$Change\, in\, {R}_{m}= \frac{{R}_{mx}-{R}_{m0}}{{R}_{m0}}\times 100 \%$$

where R_mx_ is the recorded membrane resistance of neuron recorded at trial x (1–3), R_m0_ is the membrane resistance of the neuron recorded at the first trial.

### In vivo electrophysiology

Surgery and recordings were performed under isoflurane anesthesia (3–4% induction and 1–2% maintenance). The body temperature was maintained between 37 and 38 °C using a closed loop system with a rectal probe and heating pad (DC Temperature controller, FHC). Mice were implanted with a lightweight metal head holder to the skull with cyanoacrylate glue (Loctite 401). A recording chamber from dental cement (Paladur) was made above the barrel cortex. A small craniotomy (< 1 mm) was made over the center of the primary somatosensory barrel cortex at stereotactic coordinates –1.2 mm posterior/3.5 mm lateral to bregma and the durotomy was performed to enable electrode entry.

Mice were placed under a custom made two-photon laser scanning microscope and the region of interest was scanned with a tunable mode-locked Ti:sapphire laser beam (Ultra II, Coherent) using a 16 × 0.8 NA water immersion objective (Nikon). One pipette, filled with intracellular solution containing Alexa-594 (Invitrogen), was inserted into the brain with positive pressure applied by the LN-PCS system (250 mBar; LN-PCS Luigs & Neumann). Cells were visually identified as dark areas, or ‘shadows’ against the background neuropil^31,32^. After each recording, a z-stack of a series of optical sections separated by 2 μm was created to assess the anatomy of the recorded neuron.

*Whole-cell* patch-clamp recordings were conducted using borosilicate glass capillaries with an external diameter of 2 mm (Hilgenberg) and pipettes ranging from 7 to 13 MΩ. The pipettes were filled with an intracellular recording solution containing (in mM): 135 K-gluconate, 4 KCl, 10 HEPES, 10 phosphocreatine, 4 Mg-ATP, 0.3 Na_2_-GTP (adjusted to pH 7.3 with KOH), 0.2% biocytin, and 30 µM Alexa-594 (Invitrogen).

The brain was covered with Ringer’s solution containing (in mM): 135 NaCl, 5 KCl, 5 HEPES, 1.8 CaCl_2_, 1 MgCl_2_. An Ag/AgCl ground electrode was placed in the recording chamber. The recording pipette was lowered toward the surface of the brain of interest using a motorized micromanipulator with submicrometer precision (Junior, Luigs & Neumann). As soon as the pipette penetrated the brain’s surface, the pressure was decreased to 130–150 mBar and advanced slowly to the region of interest, about 150 µm under the brain surface. The pressure was then decreased to 30 mBar during the targeting phase.

To measure the pipette resistance, a resistance test comprising voltage steps of U = 10 mV at 100 Hz was continuously applied to the electrode in voltage clamp mode. In the bath, with no physical obstruction at the pipette tip, the resulting resistance represents the pipette's inherent resistance to the flow of current (R_p_). We averaged 20 consecutive voltage steps to minimize the effects of noise. The current (I) was measured as the voltage reported by the amplifier and converted into current using the amplifier's conversion coefficient. Applying Ohm’s law (U = R_p_ × I), we determined the pipette resistance as R_p_ = U/I.

After establishing a physical contact between the pipette and the cell, releasing the positive pressure (30 mBar) induced the cell membrane to typically fuse onto the glass pipette, leading to an increase in resistance. Then, after a few seconds, a high R_GS_ forms (> 1 GΩ). Following formation of the gigaseal, we delivered 20 voltage injection steps in voltage clamp mode (10 mV at 100 Hz) and determined the seal resistance using Ohm's law.

Once we broke the seal and entered *whole-cell* configuration, we switched to current clamp and measured the input resistance of the recorded neurons using a series of hyperpolarizing current injection steps (–100 pA, 100 ms, at 4 Hz). The resulting membrane potential response to the current steps is a function of the cell's access resistance and input resistance. The cell's access resistance was estimated by taking the point of intersection between the fit of the membrane voltage (V_m_) deflection amplitude taken 2 ms after the start to the end of the current step and the vertical drop at the start of the current pulse. The input resistance was calculated from the difference in mV between the current injection response, corrected for access resistance, and the resting V_m_.

Pyramidal-like cell somata were targeted for recording using the shadow-patching technique^31,32^. The resistance of the pipette was constantly monitored on a TDS2024C oscilloscope (Tektronix). After contact was established, negative pressure was applied to form a gigaseal. Following gigaseal formation, brief, negative pressure pulses were used to break the membrane and enter *whole-cell* configuration in voltage clamp mode.

Current clamp recordings were then made using an Axon Multiclamp 700B amplifier (Molecular Devices). Recordings were digitized at 20 kHz by the analog–digital converter, Power1401 (CED; Cambridge Electronics Design), high-pass filtered at 10 kHz, and continuously collected using the Spike2 software (CED). Both pipette resistance (R_p_) and seal resistance (R_s_) were monitored during the approach and the seal formation, respectively. For successful recording with a downstate membrane potential < – 50 mV, a firing pattern protocol was applied with current step injections-(– 200 to + 300 pA in 100 pA steps). Subsequently, a protocol of hyperpolarizing current step injections was performed to monitor the cell input resistance (R_in_). Liquid junction potential was not compensated.

### Whisker stimulation

To stimulate all whiskers, we used light Airpuff stimuli. Airpuffs were delivered at 0.25 Hz through a plastic tube (3 mm diameter, 5 cm away from the whisker pad), using a solenoid valve (The Lee Company; 6 p.s.i.) controlled by Spike2 software (CED).

### Scanning electron microscopy

We prepared the pipettes for SEM imaging similar as previously reported by others^21^. The internal was removed from the pipette using a micropipette with Eppendorf epTIPS (20 µl). Then they were tip-filled with milli-Q water for 60 s by applying a strong vacuum (−300 mBar) to dilute any remaining internal solution at the tip, which could create salt crystal and compromise the imaging. Pipettes were placed to dry in a desiccator overnight and imaged the following day with a GeminiSEM 300 SEM by Carl Zeiss Microscopy at 4 keV electron energy using secondary electrons. Prior to the analyses, the pipettes were coated with Carbon to avoid charging.

### Data analysis

Data were analyzed with Matlab (version 2022a) or Stimfit 0.15 (Christoph Schmidt-Hieber, UCL) and appropriate statistical analysis were performed using GraphPad Prism 8. Representations in Figs. 1a, c and 3a were created with BioRender.com.

For in vivo electrophysiology, data analysis was carried out using a custom-made Python script. All data are plotted as the mean ± standard deviation (SD). 'N' represents the number of mice used, and 'n' represents the number of recorded neurons.

### Supplementary Information

Supplementary Information.

Supplementary Video 1.

Supplementary Video 2.
