# Supplementary material for: Immediate reuse of patch-clamp pipettes after ultrasonic cleaning
Source: Sci Rep. 2024 Jan 18;14:1660. doi: 10.1038/s41598-024-51837-7 (PMC10796327; doi:10.1038/s41598-024-51837-7)
Supplement: Supplementary file 1 — Supplementary Information. [file 41598_2024_51837_MOESM1_ESM.docx]

**Immediate reuse of patch-clamp pipettes after ultrasonic cleaning**

Kevin Jehasse^1,*^, Jean-Sebastien Johanneau^5,6^, Sophie Wetz^1,4^, Alexander Schwedt^2^, James Poulet^5,6^, Peter Neumann-Raizel^7,#^*,* Björn M. Kampa^1,3,4,#,*^

Supplementary informations

*Correspondence: Kevin Jehasse: [jehasse@bio2.rwth-aachen.de](mailto:jehasse@bio2.rwth-aachen.de); Björn M. Kampa: [kampa@brain.rwth-aachen.de](mailto:kampa@brain.rwth-aachen.de)

# equal contribution


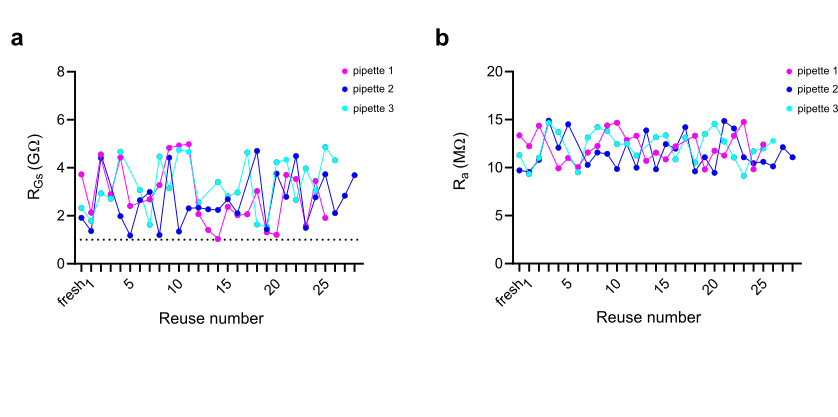


**Supplementary Figure 1: Ultrasonic cleaning allows the reuse of patch-clamp pipettes over 25 times. (a)** shows the reuse of 3 pipettes for which R_GS_ was successfully reached over multiple cleaning procedures. Success rate is 23/25 trials for pipette 1, 27/28 for pipette 2 and 24/26 for pipette 3. **(b)** shows that R_a_ is not affected by ultrasonic cleaning, in favor of indefinite reuse of the pipette. Success for whole-cell achievement is the same as **(a)**.


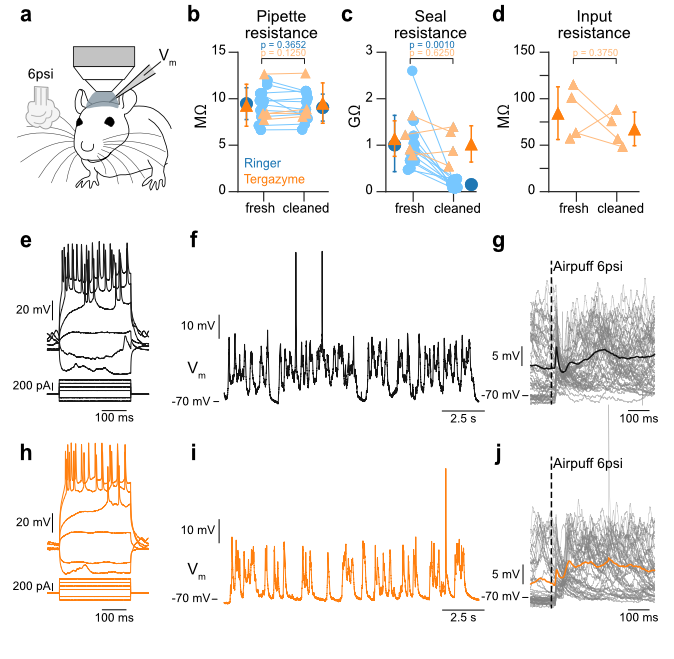


**Supplementary Figure 2: *In vivo* whole-cell membrane potential recordings. (a)** Cartoon schematic showing *in vivo* whole-cell recording setup with airpuff stimulus delivered to the whiskers and whole cell pipette in primary somatosensory barrel cortex. **(b)** Resistance of pipette in the bath before entering the brain (fresh) and after having been used to record neuronal activity and followed the LN-PCS cleaning procedure (cleaned). Blue shows data from pipettes cleaned in Ringer’s solution (N = 6 mice, n = 9 cells) or external Ringer’s (N = 2 mice, n = 2 cells) or external double distilled water (N = 1 mouse, n = 1 cell), orange shows data from pipettes cleaned using the LN-PCS system in 1% Tergazyme in double distilled water (N = 4 mice, n = 4 cells). Light colored circles and triangles connected by lines represent data from a single pipette before and after cleaning, dark color circles and triangles with error bars show mean +/- SD for each condition (Ringer’s fresh vs. cleaned: 9.40 +/- 1.62 MΩ vs 8.98 +/- 1.38 MΩ, n = 11, p = 0.3652; Tergazyme fresh vs. cleaned: 9.23 +/- 1.95 MΩ vs 9.46 +/- 1.87 MΩ, n = 4, p =0.1250). **(c)** Same plot as **(b)** but for seal resistance (Ringer’s fresh vs cleaned: 1.03 +/- 0.57 GΩ vs 0.15 +/- 0.08 GΩ, n = 11, p = 0.0010; Tergazyme fresh vs cleaned: 1.13 +/- 0.33 GΩ vs 1.02 +/- 0.34 GΩ, n = 4, p = 0.6250). **(d)** Comparison of input resistance for successful recording in Tergazyme cleaned pipettes. Light color triangles connected by lines represent data from a single pipette before and after cleaning, dark colored triangles with error bars show mean +/- SD for each condition (Tergazyme fresh vs cleaned: 83.84 +/- 24.44 MΩ vs 66.99 +/- 15.59 MΩ, n = 4, p = 0.3750). **(e)** Firing pattern from example neuron recorded with a fresh pipette. Upper panel shows the membrane potential (V_m_) of the recorded neuron responding to the 6 current steps shown on the lower panel. **(f)** Spontaneous membrane potential fluctuations from the recorded neuron shown in **(e)**. **(g)** Airpuff evoked response from recorded neuron shown in **(e)** and **(f)** with single trials shown in grey and mean response in black. **(h)**, **(i)**, and **(j)** same as **(e)**, **(f)**, and **(g)** but after cleaning with Tergazyme.

**Supplementary Video 1: Resonance effect during ultrasonic cleaning.** The resonance effect is observed on the tip of the pipette (left) during the ultrasonic cleaning of a second pipette (right) at 1 mm from it. White line shows when the cleaning starts.

**Supplementary Video 2: Absence of resonance effect during ultrasonic cleaning.** The resonance effect on the tip of the pipette (left) is absent when the ultrasonic cleaning of a second pipette (right) occurs at 5 mm from it. White line shows when the cleaning starts
